# High diagnostic yield despite a lower completion rates for inpatient versus outpatient colon and pan-intestinal capsule endoscopy: a nested case-control study

**DOI:** 10.1186/s12876-022-02561-x

**Published:** 2023-03-09

**Authors:** Charlene Deane, Caroline Walker, Barbara Ryan, Anthony O’Connor, Sarah O’Donnell, Niall Breslin, Deirdre McNamara

**Affiliations:** 1 Department of Gastroenterology, Tallaght University Hospital, Tallaght, Dublin, 24 Ireland; 2 TAGG Research Centre, School of Medicine, Trinity College Dublin, Dublin, Ireland

**Keywords:** Capsule endoscopy, colon capsule endoscopy, pan-intestinal capsule endoscopy

## Abstract

**Background:**

Increased familiarity with capsule endoscopy (CE) has been associated with a growing demand for urgent inpatient procedures. Limited data exists comparing the effect of admission status on colon capsule (CCE) and pan-intestinal capsule (PIC) performance. We aimed to compare the quality of inpatient versus outpatient CCE and PIC studies.

**Methods:**

A retrospective nested case-control study. Patients were identified from a CE database. PillCam Colon 2 Capsules with standard bowel preparation and booster regimen were used in all studies. Basic demographics and key outcome measures were documented from procedure reports and hospital patient records, and compared between groups.

**Results:**

105 subjects were included, 35 cases and 70 controls. Cases were older, were more frequently referred with active bleeding and had more PICs. The diagnostic yield was high at 77% and was similar in both groups. Completion rates were significantly better for outpatients, 43% (*n =* 15) v’s 71% (*n =* 50), OR 3, NN3. Neither gender nor age affected completion rates. Completion rates and preparation quality were similar for CCE and PIC inpatient procedures.

**Conclusion:**

Inpatient CCE and PIC have a clinical role. There is an increased risk of incomplete transit in inpatients, and strategies to mitigate against this are needed.

## Background

Colonoscopy quality is reduced when performed on inpatients for several reasons [1–5]. Where possible, many centres now advocate deferring inpatient colonoscopies in preference for an early outpatient procedure. Colon capsule endoscopy (CCE) is a viable alternative to colonoscopy in various clinical settings [6, 7]. The use of double-headed capsules with extended battery life has also been explored as a means of a pan-intestinal examination of the gastrointestinal (GI) tract. A recent systematic review confirmed that pan-intestinal capsule studies with either colon capsules or specifically designed pan-intestinal capsules are a feasible technique [8]. More recently, an Italian study confirmed that pan-intestinal assessment using colon capsules (PillCam®Colon 2) was an effective investigation strategy in patients with melena [9]. Increased awareness and familiarity with all forms of capsule endoscopy have been associated with a growing demand for urgent inpatient procedures, including colon capsules, combined colon & small bowel (SB), and pan-intestinal studies (PIC). These procedures, similar to standard colonoscopy, rely on adequate bowel preparation and GI motility, which could all be adversely affected in hospitalised patients. Currently, limited data compares the effect of admission status on CCE and PIC quality and outcomes.

## Methods

This study aimed to compare the quality of inpatient (hospitalised) versus outpatient CCE colonic and PIC studies, and to assess factors affecting the outcome.

We performed a retrospective nested case-control study at Tallaght University Hospital, Dublin, Ireland over 1 year. Adult patients who had undergone either an inpatient CCE or PIC were identified from a capsule database. Controls and subjects who had undergone outpatient CCE and PIC procedures during the same period, were sequentially selected, i.e. the next outpatient procedures after the case, in a 1:2 ratio. All participants were ambulatory and able to swallow the capsule. All procedures were performed using PillCam®Colon 2 Capsules (Minneapolis, MN, USA) using a standard bowel preparation and booster regimen. For PIC, the SB sleep mode was manually deselected prior to capsule ingestion. Risk factors for delayed transit were identified at pre-assessment for all outpatients, and if present, patients had a gastric transit assessment at 30 minutes, as per ESGE Technical Review guidance [1, 10]. Similarly, all inpatients underwent a gastric transit assessment as they are an identified at-risk group. If gastric transit was delayed and in the absence of contra-indications, patients received a prokinetic (metoclopramide 10 mg PO / IV).

Patients took 4 7.5 mg Senna tablets 2 days before the procedure. Then, the evening before the procedure, they ingested the first litre of a two-litre split-dose bowel preparation with Moviprep® (Norgine, Amsterdam, Nederland) a PEG-based solution. The second litre was taken on the morning of the procedure and all procedures were performed before 12:00. The first booster, Moviprep® with 750 ml of water and 15 ml of castor oil, was given when the capsule reached the small bowel. Then, 3 hours later a second booster of Moviprep® with 250 ml of water was given [11].

All studies were analysed by trained capsule endoscopists using Rapid Reader software version 9.0, and the findings were approved by our institution’s capsule review board. Basic demographics and key outcome measures were identified from the procedure reports and hospital patient records as required. Findings were compared between groups using _X_ [2] or student t-tests as appropriate, and relevant odds ratio (OR) calculations were performed as indicated. A *p*-value of < 0.05 was considered significant.

## Results

In total, 105 subjects were included; 35 cases (inpatient procedures) and 70 controls (outpatient procedures). Inpatients were older (72 vs 56 years, *p <* 0.0001), more frequently had a PIC (29 (83%) versus 11 (16%), *p <* 0.0001), and were referred more often for investigation of GI bleeding, either overt or occult, than controls, 28 (80%) versus 20 (29%), *p <* 0.0001), (Table 1).

**Table 1:** Study population; CCE: colon capsule endoscopy; PIC: pan-intestinal capsule

	Inpatient Cohort *N =* 35	Outpatient Cohort *N =* 70	Significance
Mean Age in Years	72.4	56.6	*P <* 0.0001
Male Gender N (%)	20 (57%)	31 (44%)	NS
Indication for Capsule N (%)			
*GI Bleeding*	28 (80%)	20 (29%)	*P <* 0.0001
*Polyp Surveillance*	1 (3%)	19 (27%)	*P <* 0.007
*Symptoms- Diarrhoea / Abdominal pain*	3 (9%)	17 (24%)	NS
*Other*	3 (9%)	14 (20%)	NS
Procedure Type N (%)			
CCE	6 (17%)	59 (84%)	*P <* 0.0001
PIC	29 (83%)	11 (16%)	*P <* 0.0001

Completion rates defined as the capsule passing the dentate line were significantly lower in inpatients, 43% (*n =* 15) compared to controls 71% (*n =* 50), *p <* 0.0007, OR 3, 95%CI 1.59-8.79. Neither age nor gender affected completion rates. However, more inpatients had one or more risk factors for slow transit *n =* 21 (60%) versus 4 (6%) of outpatients, *p <* 0.0001, OR 24.7, 95% CI 7.3444 to 83.4050. (Table 2).

**Table 2:** Completion rates and associated factors by cohort

	Inpatient Complete *N =* 15	Inpatient Incomplete *N =* 20	Outpatient Complete *N =* 50	Outpatient Incomplete *N =* 20
Mean Age in Years	82.5	71	54.5	65
Gender				
Male / Female	8/7	11/9	28/22	9/11
Bowel Preparation				
*Adequate*	15 (100%)	9 (45%)	46 (92%)	13 (65%)
*Inadequate*	0 (0%)	11 (55%)	4 (8%)	7 (35%)
Delayed Transit Risk Factor Positive	21 (60%)		4 (6%)	

Bowel preparation quality was similar between groups, defined as adequate (Boston Bowel Preparation Score ≥ 5) or better in 24 (69%) and 59 (84%) of inpatient and outpatient studies, respectively. As expected, though, complete studies were associated with adequate bowel preparation 61/65 (94%) complete vs 22/40 (55%) incomplete CE studies, *p <* 0.0001, OR 12, 95% CI 3.8033 to 40.933. Notably, bowel preparation, completion rates, and diagnostic yield were similar for both CCE (*n =* 65) and PIC (*n =* 40) procedures. (Table 3).

**Table 3:** Colon capsule endoscopy (CCE) and pan-intestinal capsule (PIC) endoscopy performance

	PIC *N =* 40	CCE *N =* 65
Adequate Bowel Preparation	29 (73%)	54 (83%)
Complete Study N (%)	20 (50%)	45 (69%)
Diagnostic Yield N (%)	31 (78%)	49 (75%)

Despite the difference in completion rates, the diagnostic yield was similar in both inpatient and outpatient cohorts, 80% (28/35) and 74% (52/70), respectively. Diagnostic yield was not affected by indication, being similar for both bleeding 73% (35/48) and all other indications 79% (45/57). However, for those presenting with bleeding as an indication the yield was higher, 79% (22/28) in inpatients compared to 35% (7/20) in outpatients, *p <* 0.002, OR 6.8, 95%CI 1.87- 24.69. Also more patients in the inpatient cohort were diagnosed with vascular lesions 6 (17%) versus 1 (1.4%); likely reflecting the indication of bleeding bias for that cohort. Table 4. Overall, there were no technical issues with video capture and there were no procedure related complications.

**Table 4:** Capsule performance by admission status

	Inpatient Cohort *N =* 35	Outpatient Cohort i70	Significance
Complete Study N (%)	15 (43%)	50 (71%)	*P <* 0.0001
Adequate Bowel Preparation N (%)	24 (69%)	59 (84%)	NS
Diagnostic Yield N (%)	28 (80%)	52 (74%)	NS
Findings N (%)			
Colonic Polyps	17 (49%)	27 (39%)	NS
Vascular Lesions	6 (17%)	1 (1%)	*P <* 0.008
Diverticulae	9 (26%)	25 (36%)	NS
Inflammatory Bowel Disease	1 (3%)	10 (14%)	*P<*
Ulceration (NSAID)	2 (6%)	1 (1%)	
Other	3 (9%)	4 (6%)	

## Discussion

To our knowledge, this is the first study to report on the efficacy of colon capsule and pan-intestinal capsule endoscopy in an inpatient cohort compared to day case procedures. There is now evidence to show that colonoscopy quality is lower in hospitalised patients, with lower preparation quality and higher rates of incomplete examinations. As CCE and PIC, in keeping with colonoscopy, rely on adequate bowel preparation and, more so, on good gut motility, we surmised that both CCE and PIC performance would be adversely affected in an inpatient cohort. As expected, completion rates were significantly lower in inpatients than outpatients, 43% versus 71%, OR 3, *p <* 0.001. While more outpatient procedures had an adequate or better bowel preparation, 84% versus 69%, this did not meet statistical significance. As expected, though, complete studies were associated with adequate bowel preparation 61/65 (94%).

Despite this, the overall diagnostic yield was high and similar in both cohorts (inpatients 80% and outpatients 74%). This is not surprising as most inpatients were referred for PIC or CCE with suspected bleeding (80%). The link between increased capsule endoscopy yield and procedure timing in patients with suspected SB bleeding has been well established. The European Society of Gastrointestinal Endoscopy (ESGE) recommends that SB CE be performed within 14 days of a bleeding episode to enhance yield, while more recent studies suggest an even shorter interval is optimal [12–14]. Additionally, earlier use of SB CE in inpatients with suspected bleeding after a negative upper GI endoscopy may shorten inpatient stays and decrease the need for colonoscopy [15]. Although the data is unavailable, our inpatient procedures were likely, by their nature, performed soon after the bleeding event, optimising yield for colonic and small bowel lesions alike, despite the lower completion rates and tendency towards poorer bowel preparation.

It is well-recognised in CE practice that certain groups are at risk of delayed gastric and small bowel transit, with real-time assessment of passage advised in at-risk patients for small bowel studies and an automated built-in alert system employed in all subjects undergoing CCE [13]. Indeed, previous studies have confirmed an increased risk of delayed gastric transit and incomplete studies when small bowel capsules are performed in an inpatient setting [16, 17]. Many inpatient factors include reduced mobility, more frequent comorbidities, and medication use cited as potential causes. In keeping with this, more of our inpatient cohort had at least one factor for delayed transit (60%). In our cohort, we routinely checked for delayed gastric transit at 30 minutes. We administered a prokinetic if passage into the SB was not confirmed in both PIC and CCE studies. In addition, all subjects additionally received two standard PEG-based boosters on reaching the small bowel and 3 hours later, to promote transit through the colon. Despite this, completion rates for both CCE and PIC studies remained lower in our inpatient compared to the outpatient cohort. A tailored inpatient regimen should be considered to mitigate against this, such as additional real-time transit checks and timed boosters and prokinetics related to the stage of transit rather than from the time of ingestion and passage into the SB, as well as avoiding pro re neta medications which could adversely affect the study outcome and encouraging motility where possible. Such interventions warrant additional investigation as our study suggests that despite the limitations, inpatient CCE and PIC are useful investigations.

The role of capsule endoscopy as a pan-intestinal investigation is expanding, with evidence to show efficacy for several indications, including bleeding, enteropathy and Crohn’s disease assessment [9, 18, 19]. Both double-headed colon capsules and dedicated pan-intestinal capsules (PICE) have been used for pan-intestinal investigation. While the software design for PICE may be advantageous in assessing Crohn’s disease, colon capsules appear to have similar efficacy for pan-intestinal investigation. A recent systematic review included 357 and 424 cases employing newer colon capsules and pan-intestinal capsules, respectively [8]. Of 16 studies included, 13 were in patients with Crohn’s disease. They reported both cleanliness and completeness rates were acceptable in all studies, ranging from 63.9 and 68.6% to 100% [8] . Our study also suggests PIC with CCE is effective in various clinical settings, with a diagnostic yield of 78%.

Our study has several limitations; its retrospective design and inherent bias are unavoidable. The real-world setting and relatively large numbers of unselected inpatient PIC cases help assist practitioners in determining the true role of urgent PIC and CCE investigations in a clinical setting and in guiding further investigations to optimise performance.

## Conclusion

Inpatient pan-intestinal and CCE have a clinical role, particularly in the setting of acute bleeding. However, practitioners should be aware of the increased risk of incomplete capsule transit and develop additional techniques to mitigate this where possible.

## Data Availability

All data generated or analysed during this study are included in this published article.

## Abbreviations

CCE: Colon capsule endoscopy
CE: Capsule endoscopy
CI: Confidence interval
ESGE: European Society of Gastrointestinal Endoscopy
IV: Intravenous
ml: Millilitres
OR: Odds ratio
PIC: Pan-intestinal capsule
PO: Per os / by mouth
